# Side Effects in Time Discounting Procedures: Fixed Alternatives Become the Reference Point

**DOI:** 10.1371/journal.pone.0165245

**Published:** 2016-10-21

**Authors:** Przemysław Sawicki, Michał Białek

**Affiliations:** Kozminski University, Centre for Economic Psychology and Decision Sciences, Jagiellonska 59, Warsaw 03–301, Poland; Technion Israel Institute of Technology, ISRAEL

## Abstract

Typical research on intertemporal choice utilizes a two-alternative forced choice (2AFC) paradigm requiring participants to choose between a smaller sooner and larger later payoff. In the adjusting-amount procedure (AAP) one of the alternatives is fixed and the other is adjusted according to particular choices made by the participant. Such a method makes the alternatives unequal in status and is speculated to make the fixed alternative a reference point for choices, thereby affecting the decision made. The current study shows that fixing different alternatives in the AAP influences discount rates in intertemporal choices. Specifically, individuals’ (*N* = 283) choices were affected to just the same extent by merely fixing an alternative as when choices were preceded by scenarios explicitly imposing reference points.

## Introduction

In a landmark study, Mischel et al. [1] presented four-year-old children with a marshmallow and told them that if they waited fifteen minutes they would receive two instead of just the one initially offered. Some children resisted the temptation of immediate consumption while others did not. The former fared better in follow-up studies measuring their Scholastic Aptitude Test (SAT) performance, ability to cope with personal problems, etc., and had more healthy body mass indices (BMI). In another study, Moffitt et al. [2] showed that, as adults, more impatient children tend to be less healthy, more obese, have worse financial and working situations, and tend to be less happy in their romantic relationships. The above research suggests that knowledge of people’s ability to delay gratification can be a good predictor of their future wellbeing.

Delaying gratification, as done in the marshmallow test, constitutes a simple one-shot choice which can be studied in a natural environment. However, this does not permit the cognitive processes underlying a decision to be controlled [3]. Therefore behavioral researchers have developed more sophisticated methods to allow calculation of people’s discount rates. The term discount rate describes people’s ability to delay payoffs: a higher discount rate indicates a weaker ability to delay payoffs and a lower discount rate indicates a stronger ability to delay payoffs.

The simplest technique among these methods is the matching choice method: a person is explicitly asked to indicate an amount that they would want to receive in the future as the equivalent of a present given amount, e.g., *what is the least amount you would want to receive in 3 months instead of having $100 now*? [4,5]. A variation of this method is to ask people to select the expected amount from a drop-down menu [6].

Another group of methods involves the two-alternative forced choice paradigm (2AFC), in which people select between smaller sooner (SS) and larger later (LL) payoffs. Of these two alternatives, one is fixed and the other varies. The most prominent methods used are the staircase method and the adjusting amount procedure (AAP) [7]. In the staircase method, the varying alternative is presented in ascending or descending order, and the procedure stops when a person changes their preference from one alternative to the other [8,9]. For example, if considering the delayed equivalent of $5,000 (SS), a person decides between this and LL—$5,100. If they decide to take LL, the procedure stops, if they decide in favor of SS, LL increases, e.g., to $5,200. The indifference point for SS and LL is the mean of the two last steps, i.e. $5,050 if the staircase ends at the first step, $5,150 if it ends at the second, etc. The order of presentation of the varying alternative has a significant impact on discount rates[10].

In the AAP, the varying amount is adjusted according to choices made by the participant [11–13]. For example, where a researcher aims to calculate the indifference point for LL, the first step requires a choice between SS and LL, where SS is typically equal to half of LL. Choosing LL increases the value of SS in the next step, but choosing SS decreases its value in the next step. The degree of adjustment decreases by a half in consecutive steps until the difference is acceptably small (so called granularity [14]). The SS-LL indifference point is the mean of the last two steps.

All of the above methods are used repeatedly for different delays and provide a set of indifference points. These indifference points serve as a basis for calculating discount rates. There are two methods of doing this: fitting the points to a curve using either a hyperbolic or exponential formula [15,16], or calculating the area under the curve (AUC) [17]. We will use the latter, as it is assumption free, and will provide its detailed description in the next paragraphs.

A common feature of all the mentioned methods is that one option is fixed and the other is determined by the participant, and this unequal status of alternatives can have an impact on participants’ choices. The aim of the present work was to investigate this impact experimentally. We tested the idea that the unequal status of alternatives can be confounded with certain experimental manipulations, such as imposing a temporal reference point.

Typical methods used to explore the factors affecting discount rates use scenarios which precede the method of calculating discount rates, and which explicitly impose a reference point. Reference points affect human choices in all domains, not only in intertemporal choices. An illustrative example is that in Poland it is legal to harvest someone’s organs after their death and transplant them unless they have previously declared otherwise. Only a small percentage of Polish citizens declare their refusal (there are about 26,000 registered declarations in a country of 38 million people, [18]). In contrast, US law requires a potential donor to make a clear statement of their agreement, with only 10% of people making such a declaration [19,20]. This striking difference results from differences in the presentation of options and from imposing different default actions. These presentational differences can serve as methods for influencing peoples’ choices [21,22].

As previously mentioned, reference points are important in research on discounting. For example, when investigating the so called direction effect, participants are asked to imagine: (a) that they are expecting some future income and declare the minimum acceptable amount that they would want to receive it immediately, or (b) that they will receive some income immediately, and declare what the minimum premium would be for them to wait. The first scenario imposes a reference point in the future (an accelerating scenario) and the latter scenario imposes a reference point in the present (a delaying scenario). Discount rates obtained in studies are higher when participants focus on the present compared to when they focus on the future, this emphasizing the significance of reference points for subsequent choices [4,6,23].

In experiments investigating the direction effect, experimental conditions differ not only by scenario type (accelerating/delaying) but also by the experimental procedure employed. Specifically, when a decision problem is framed using a reference point in the future, an accelerating scenario is presented and the LL alternative is fixed while SS is adjusted. However, when a problem is framed using a reference point in the present, a delaying scenario is presented and the SS alternative is fixed while LL is adjusted [4,9].

We hypothesized that a fixed alternative becomes a reference point for consecutive choices and affects the discount rates obtained. More specifically, we reasoned that the unvarying alternative can affect subsequent choices because participants can think of the task as a game in which they need to receive as much as possible for what they currently have, e.g., to obtain the maximum premium possible for waiting six months instead of receiving a promised $5, 000 immediately, or to lose as little as possible for accelerating an expected future payoff. Such framing of a choice, compared to a neutral choice between two payoffs differing by amount and time of receipt, can result in stronger discounting for delaying income (maximizing a bonus) and weaker discounting for accelerating income (minimizing a reduction in gain). Such an effect is consistent with the direction effect, but obtained without using any explicit manipulation of a reference point.

### The present experiment

To test the impact of a fixed alternative on discount rates it is necessary to remove the effects of scenario type. To achieve this, we compared neutral scenarios with scenarios imposing a reference point, each followed by the adjusting-amount procedure (AAP). We used the AAP because it is currently one of the most commonly used methods. However, the AAP is just one example in a whole category of procedures where one alternative is fixed and the other is provided by participants, and which share the same feature of unequal status of alternatives.

We expected to replicate findings on the direction effect obtained using classical accelerating/delaying scenarios followed by the AAP. We also expected to observe similar effects by merely fixing one of the alternatives (thus making it more prominent compared to the adjusted alternative) and using a neutral scenario which did not impose a reference point. As the fixed alternative was expected to become the reference point, fixing the LL alternative was expected to create a reference point in the future, and fixing the SS alternative was expected to create a reference point in the present.

To increase the reliability of findings we ran the experiment in the domains of both gains and losses, expecting the effect of fixing an alternative to be observed in both domains.

We confirm, that in this work, just as in all our other publications, we have reported all measures, conditions, data exclusions. We also explained how we determined the sample size.

## Method

### Participants

The initial sample of participants consisted of 300 native English speakers from the USA, UK and Canada, enrolled from Amazon’s MTurk worker pool. The number of participants (around *n* = 35 for each condition) was guided by sample sizes in previous experiments investigating the direction effect [24–26]. All participants were financially compensated for their time (each being paid a fee of $2.45 for their participation) and gave written informed consent. The study was approved by the Kozminski University Ethics Committee and carried out in accordance with approved guidelines.

Prior to data analysis, cases where indifference points did not monotonically decrease or increase with time were identified [27]. We excluded data for these individuals as they: (1) might have made a mistake in selecting an incorrect alternative, or (2) had a negative discount rate, which can be assumed to be qualitatively distinct from classical discounting [28] and was therefore beyond the scope of this paper. After employing the exclusionary criterion, the final sample consisted of 283 participants (43% female, *M* age = 34 years, *SD* = 12.4, range 18–74).

A between-subjects design was used to exclude the possibility of any interaction between conditions. Thus, each participant was assigned to one of eight experimental groups: 2 sign (gains, losses) x 2 alternative fixed (SS or LL) x 2 scenario type (neutral, imposing a reference point). Each participant performed the subsequent AAP task for three delays (1, 6, and 24 months). Table 1 presents a detailed description of each experimental condition together with participants’ demographic details.

**Table 1:** Descriptions of each condition in the experiment.

Condition No.	Alternative fixed in AAP	Scenario type	Sign	*n*	No. of females	Mean age	*SD* Age
1	LL	ACC	Gains	49	17	32.9	12.2
2	LL		Losses	26	10	36.8	13.1
3	SS	DEL	Gains	38	22	37.5	11.5
4	SS		Losses	49	20	35.3	9.8
5	LL	NEU	Gains	23	13	36.0	14.0
6	LL		Losses	26	10	32.6	12.4
7	SS		Gains	37	11	32.0	9.3
8	SS		Losses	35	18	36.3	14.1
SUM				283	121	34	12.4

SS–smaller latter, LL–larger latter, ACC–accelerating, DEL–delaying, NEU—neutral

### Scenarios

In half of the groups, participants read a scenario intended to impose a reference point (accelerating/delaying scenarios) while the other half were presented with a neutral scenario. The neutral scenarios were designed so as to not make any of the considered alternatives more prominent, while the other scenarios focused participants on a particular alternative. The scenarios described either a situation involving a gain (inheriting $5, 000) or a loss (receiving a $5,000 fine), which were to be paid either immediately or with a delay. Table 2 presents the exact wording of each scenario used in the experiment.

**Table 2:** Wording of scenarios.

Scenario	GAINS	LOSSES
Delaying scenario (reference point in the present)	Imagine that you have received a $5,000 donation which will be paid to you immediately. An investment fund offers to delay depositing this money for 1 month (4 weeks) in exchange for some interest. Consider what you would do: accept or refuse the offer.	Imagine that you have received a $5,000 fine which has to be paid immediately. An investment fund offers to pay this fine for you but you will have to pay the money back in 1 month (4 weeks) in exchange for some interest. Consider what you would do: accept or refuse the offer.
Accelerating scenario (reference point in the future)	Imagine that in 1 month (4 weeks) you will receive a donation of $5,000. An investment fund offers to accelerate the donation but the payment will be smaller. Consider what you would do: accept or refuse the offer.	Imagine that you have received a $5,000 fine, but you need to pay it in 1 month. An investment fund offers to take over this obligation if you pay a certain part of the fine to them immediately. Consider what you would do: accept or refuse the offer.
Neutral scenario (no reference point)	Imagine that you have inherited a specific sum of money. It can be paid to you in one of two ways: (A) You will receive the money with a delay but the amount will be greater, (B) You will receive the money immediately but the amount will be lower. Consider which payment option you would choose.	Imagine that you have received a fine which you can pay in one of two ways: (A) You will pay the fine with a delay but the amount will be greater, (B) You will pay the fine immediately but the amount will be lower. Consider which payment option you would choose.

### The Adjusting Amount Procedure (AAP)

After reading one of the three scenarios, participants performed an adjusting-amount procedure (AAP) to determine their discount rates. In this procedure an individual chooses between two alternatives: a smaller sooner (SS) or a larger later (LL) alternative, e.g., receiving $5,000 immediately (SS) or $7,500 in 3 months (LL). In a set of choices one of the alternatives is fixed and the other is adjusted according to previous choices. The details of the adjusting-amount procedure are presented in Table 3.

**Table 3:** The adjusting-amount procedure. A general description of the adjusting-amount procedure used in the experiment. Reference points were provided by the fixed alternative.

Fixed LL alternative (reference point in the future):	Fixed SS alternative (reference point in the present):
The goal was to calculate the immediate equivalent of a delayed $5,000. The first choice presented to a participant was between $2,500 to be paid immediately (SS) and $5,000 paid with a specific delay (LL). When the LL was chosen, in the following step SS increased by half of the difference between SS and LL. When the participant chose SS, the immediate value decreased by half of the difference between SS and LL. In subsequent choices the adjustment amount decreased by half of its previous value (i.e., by $675 in the second step). Thus, the immediate payoff was adjusted by half of the previous value to reflect a participant’s choices.	The goal was to calculate the delayed equivalent of $5,000 paid immediately. The first choice presented to a participant was between $5,000 to be paid immediately (SS) and $7,500 paid after a specific delay (LL). When LL was chosen, in the following step LL decreased by half of the difference between SS and LL. When the participant chose SS, the delayed value increased by half of the difference between SS and LL. In subsequent choices the amount of adjustment decreased by half of its previous value (i.e., by $675 in the second step). Thus, the delayed payoff was adjusted by half of the previous value to reflect a participant’s choices.

Each participant’s task was to choose between a SS or LL payoff. Three delays were tested: 1 month, 6 months and 24 months. Delays were presented in fixed chronological order, the delay increasing in successive trials. The AAP consisted of six steps for each delay, resulting in each participant making 18 dichotomous choices. As our interest was in the fixed (discounted) alternative, this had the same value in each condition ($5,000). However, the adjusting-amount procedure required that the adjusted alternative was different for different conditions: in the first step, when accelerating, the decision was between $2,500 immediately (adjusted) and $5,000 later (fixed); when delaying, the decision was between $5,000 immediately (fixed) and $7,500 later (adjusted).

The groups also differed according to which alternative was fixed in the AAP. Specifically, we replicated the classical procedure for participants who had read the scenarios imposing a temporal reference point: when framed for the present, the SS alternative was fixed; when framed for the future, the LL alternative was fixed. Participants who read the neutral scenarios performed the APP with random fixing of either the SS or LL alternative.

Finally, half of the participants in each condition discounted gains and the other half discounted losses.

This resulted in the creation of eight experimental groups as described in Table 1.

## Results

The area under the curve (AUC) was computed to calculate individual discount rates [17]. Because there is much discussion as to which model fits observed behavior better [29,30], we preferred AUC to the k-parameter because the former is assumption free: it does not require fitting to either an exponential or hyperbolic curve.

To calculate the AUC we scaled the difference between discounted values and the constrained end of their equivalents from 0 to 1 (for the accelerating condition, equivalents ranged $1–5,000 were scaled in the range 0–1; for the delaying condition, equivalents ranged $5,001–10,000 were scaled 0–1). The delay for each equivalent was also scaled 0–1. Each participant’s scaled indifference points were placed in a matrix and connected creating a trapezium, the area of which was calculated. Discounting rates were therefore in a range from 0 (maximal discounting strength, all delayed payoffs having a utility equal to 0) to 1 (minimal discount strength, all delayed payoffs having maximal utility).

**Fig 1:**
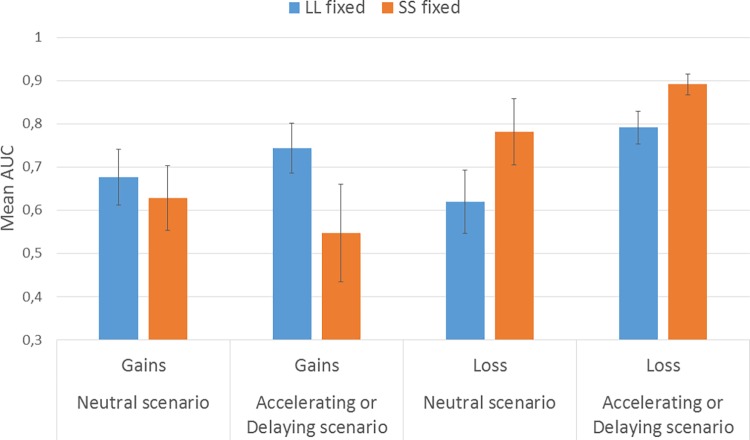
**Mean areas under the curve for the adjusting-amount procedure (AAP) preceded either by scenarios influencing the reference point (A) or by a neutral scenario (B).** A lower score indicates stronger discounting. Error bars represent 95% confidence intervals.

To test the impact of different procedures on discounting rates we conducted a 2 sign (gains, losses) x 2 alternative fixed (SS or LL) x 2 scenario type (neutral, imposing a reference point) between-subjects ANOVA. The mean AUC for each experimental group is presented in Fig 1. We replicated the classical sign effect, *F*(1,275) = 21.334, *p* < .001, ηp^2^ = .072, showing a higher discounting rate (lower AUC) for gains than losses [31].

However, our main interest lay in comparing discount rates of payoffs of the same sign preceded by different scenarios (in Table 1, conditions 1 vs. 3, and 2 vs. 4). The same differences were expected in conditions which differed only by the AAP (in Table 1, conditions 5 vs. 7, and 6 vs. 8). Main effects of the alternative fixed and scenario type were not expected as these had opposing directions of influence depending on the sign of the payoff, a so called sign by direction asymmetry [6,9,32]. Indeed, we found none of these main effects, but the sign effect interacted with the alternative fixed, *F*(1,275) = 22.894, *p* < .001, ηp2 = .077. Multiply comparisons using Bonferroni correction showed, that losses were discounted less when the SS alternative was fixed than when the LL was fixed, mean AUCs = .836 and.706 respectively, *F*(1,132) = 11.721, *p* = .001; ηp2 = .041. However, gains were discounted more strongly when the SS alternative was fixed than when the LL was fixed, mean AUCs = .587 and.710 respectively, *F*(1,143) = 11.174, *p* = .001; ηp2 = .039. The impact of the fixed alternative was the same regardless of whether the AAP was preceded by a scenario imposing a reference point or by a neutral scenario (three-way interaction *p* =. 650).

Decomposing the three-way interaction we see that the sign by direction asymmetry effect was obtained in both conditions: when the AAP was preceded by scenario imposing the reference point, *F*(1,158) = 12.782, *p* < .001; ηp2 = .075, and in neutral scenario, *F*(1,117) = 12.169, *p* = .001; ηp2 = .094. Further decomposing these interactions by multiply comparisons using Bonferroni correction, we see that the discount rates were higher for gains, when the SS was fixed, both when the AAP was preceded by a scenario imposing the reference point, (AUC_LL_ = .744, AUC_SS_ = .628; *F*(1,275) = 6.268, *p* = .013; ηp2 = .022), and by neutral scenario, (AUC_LL_ = .676, AUC_SS_ = .547; *F*(1,275) = 5.163, *p* = .024; ηp2 = .034). The discount rates were lower for losses when the SS was fixed, both when the AAP was preceded by a scenario imposing the reference point, (AUC_LL_ = .620, AUC_SS_ = .781; *F*(1,275) = 9.570, *p* = .002; ηp2 = .034), and by neutral scenario, (AUC_LL_ = .792, AUC_SS_ = .891; *F*(1,275) = 3.215, *p* = .074; ηp2 = .012). All these results are suggesting that the effect of fixing an alternative is independent of an explicitly imposed reference point

Scenario type interacted with sign of payoff, *F*(1,275) = 13.638, *p* < .001, ηp2 = .047. Multiply comparisons using Bonferroni correction showed a lower discounting rate (higher AUC) for gains when the AAP was preceded by a scenario imposing a reference point compared to a neutral scenario, mean AUCs = .686 and.612 respectively, *F*(1,275) = 4.075, *p* = .044; ηp2 = .015, but a higher discounting rate (lower AUC) for losses when the AAP was preceded by a classical accelerating/delaying scenario compared to a neutral scenario, mean AUCs = .701 and .841 respectively, *F*(1,275) = 13.638, *p* < .001; ηp2 = .047.

Summarizing, the above results show that merely fixing an alternative produces the same effects on discount rates as using scenarios which explicitly impose a reference point.

## Discussion

Identifying contextual variables that may affect discounting is an important consideration both for researchers designing experiments to investigate discounting and for practitioners. Knowledge of the impact of procedures in and of themselves allows researchers to consider these impacts when designing experiments, and facilitates comparisons across different studies and designs. This is particularly important as reported discount rates vary markedly across different studies [33]. Additionally, practitioners may be able to use the present findings to devise choice architectures which nudge individuals to make better decisions, e.g., to decrease impulsive buying, eating and promiscuity, or to increase willingness to save or buy insurance. Such nudging would not require any explicit framing, but merely changing the presentation of choice alternatives would achieve the desired effects.

The contextual variables at issue are exemplified by reference points which make one or other alternative more preferable. In previous experiments on time discounting, reference points were induced either by specifically including them, e.g., by presenting a discounting task within a specially designed scenario [34], or by priming participants for a particular reference point [35,36]. However, the present study showed that the fixed alternative in the two-alternative forced choice paradigm (2AFC) also imposes a reference point. We illustrated this by studying the adjusting-amount procedure (AAP), but this is representative of all other experimental procedures which create an unequal status between alternatives [13,33,37].

The current experiment suggests that the provided fixed alternative becomes a reference point for consecutive choices, affecting preferences. Thus, a fixed smaller sooner (SS) alternative creates a reference point in the present, and this results in a higher discounting rate for gains compared to when the reference point is in the future (where the larger later alternative is fixed). The same fixed SS alternative affects losses so that they are discounted with a lower discounting rate than when the reference point is in the future (LL fixed). Adding a scenario which imposes a reference point has no impact on these effects. This constitutes clear evidence of how a fixed alternative affects discount rates, both in the domain of gains and in the domain of losses.

The reported impact of fixed alternative cannot be explained by merely artefacts of the AAP. For example, as Białek and Sawicki [38] have shown, individuals who score high in the cognitive reflection test (CRT) show similar discount rates regardless of the imposed reference point, while those who score low in the CRT were influenced by imposed reference point and exhibit a classical DE. Indeed, we see that the AAP allows individuals to provide consistent discount rates regardless of imposed reference point, however it is more likely for individuals who reflect more.

Query Theory [39,40] provides a good account of the presented here finding. In this view, thoughts that are considered first shape preferences. When the SS alternative is fixed it is considered first and therefore people have a greater preference for the present. The same happens when the LL alternative is fixed: people consider this alternative first and have a resultant greater preference for the future. The order of queries results in greater impulsivity when the focus is on the present compared to when it is on the future [9]. This said, the concept of loss aversion- individuals being more affected by expected losses than expected gains [41,42]—can provide an alternative explanation of the findings. The perspective from which a choice is seen can move an option into another domain, i.e., delaying a gain can be seen as a loss (“*I won’t get the money now*”), hence people will be more impulsive when delaying gains than when accelerating gains (“I will get the money now”). The same applies for losses, where delaying a loss can be seen as a gain (“*I won’t lose the money now*”). Here, such framing would result in weaker discounting compared to when accelerating a loss (“*I will lose the money just now*”).

We have shown that certain variants of a commonly used method impose an implicit reference point. Currently, researchers are developing methods of measuring time discounting that are free from temporal reference points, e.g., conjoint analysis [43], the three-option adaptive discount rate measure (ToAD) [14] and randomized binary choice [44]. These methods are relatively new and are yet to gain popularity, the majority of experiments still utilizing one of the methods discussed earlier.

Our work illustrates that the older, more popular, methods all impose reference points, and sometimes more than one reference point is imposed. For example, in the research conducted here, and other research which is reviewed in the paper, the scenario and the fixed alternative both impose congruent reference points affecting the discount rates obtained. However, situations can be envisaged in which reference points are incongruent, e.g., where an individual reads a scenario imposing a reference point in the present, but the fixed alternative in the 2AFC paradigm imposes a reference point in the future (the LL alternative is fixed). We speculate that the effect of reference points would be reduced here, and that there would be a lesser influence on the discount rates obtained.

Our findings have limitations in that they result from a single study, and further studies should test the reliability of the current findings. Also, one could argue that our findings result from a confound between reference points and a magnitude effect whereby smaller amounts are discounted more strongly than greater amounts [45,46]. Indeed, the values of alternatives in the accelerating and delaying conditions were not equal ($2,500 vs. $5,000 in the accelerating condition, but $5,000 and $7,500 in the delaying condition). However, our design reduced this confound because the magnitude effect resulted from the value of the discounted alternative—which was always equal to $5,000 –and not from the other alternative [4,33]. If the adjusted amount had created the magnitude effect, stronger discounting of the smaller amount ($2.500) used in the accelerating condition than of the $7.500 used in the delaying condition would be expected. However, the results showed the opposite pattern of changes in discount rates. Of course, the fixed amount might have seemed to be greater or smaller in comparison to the varying amount, but we would expect such an effect to be negligible.

To conclude, we have confidence that our results can facilitate research on time discounting, having obtained evidence that fixed alternatives in time discounting affect the discount rates which studies obtain. Thus, the impact of indirectly imposed reference points on time discounting should be considered by researchers in their future work. For example, it would be useful to investigate problems in which combining a scenario and a fixed alternative in the AAP creates reference points which are incongruent. This incongruity could hypothetically result in reference-point free preference construction, a “clearer” insight into one’s true preferences, as these would be separated from the susceptibility to imposed reference points [38].

## Supporting Information

S1 File Supplementary material (DOCX)

S2 File baza PLOS side effects (ZIP)
