# Correction: Side Effects in Time Discounting Procedures: Fixed Alternatives Become the Reference Point

**DOI:** 10.1371/journal.pone.0176620

**Published:** 2017-04-20

**Authors:** Przemysław Sawicki, Michał Białek

In the Funding section, the Polish National Science Centre’s website is listed incorrectly. The correct website is: http://www.ncn.gov.pl.
