# Supplementary material for: Side Effects in Time Discounting Procedures: Fixed Alternatives Become the Reference Point
Source: PLoS One. 2016 Oct 21;11(10):e0165245. doi: 10.1371/journal.pone.0165245 (PMC5074525; doi:10.1371/journal.pone.0165245)
Supplement: S1 File — (DOCX) [file pone.0165245.s001.docx]

**Supplementary material**

Here, we attach the reported three-way interaction (sign by direction by scenario), in which we additionally controlled for the gender. This potential confound could have affected the reported findings. Although they are fully consistent with these reported previously, we decided to include these here. All effects had the Bonferroni correction for multiply comparison, in order to decrease the probability of type I error.

Now we show, that the impact of the fixed alternative was the same regardless of whether the AAP was preceded by a scenario imposing a reference point or by a neutral scenario (three-way interaction *F* < 1, *p* =. 703). Decomposing the three-way interaction we see that the sign by direction asymmetry effect was obtained in both conditions: when the AAP was preceded by scenario imposing the reference point, *F*(1,157) = 12.238, *p* < .001; ηp2 = .072, and in neutral scenario, *F*(1,116) = 11.728, *p* = .001; ηp2 = .092.

Further decomposing these interactions, we see that the discount rates were higher for gains, when the SS was fixed, both when the AAP was preceded by a scenario imposing the reference point, (AUC_LL_ = .742, AUC_SS_ = .629; *F*(1,274) = 5.852, *p* = .016; ηp2 = .021), and by neutral scenario, (AUC_LL_ = .678, AUC_SS_ = .545; *F*(1,274) = 5.326, *p* = .022; ηp2 = .019). The discount rates were lower for losses when the SS was fixed, both when the AAP was preceded by a scenario imposing the reference point, (AUC_LL_ = .620, AUC_SS_ = .782; *F*(1,274) = 9.574, *p* = .002; ηp2 = .034), and by neutral scenario, (AUC_LL_ = .790, AUC_SS_ = .892; *F*(1,274) = 3.294, *p* = .071; ηp2 = .012). All these results are suggesting that the effect of fixing an alternative is independent of an explicitly imposed reference point.
